# Liquid Chromatography–Tandem Mass Spectrometry for the Simultaneous Determination of Doxorubicin and its Metabolites Doxorubicinol, Doxorubicinone, Doxorubicinolone, and 7-Deoxydoxorubicinone in Mouse Plasma

**DOI:** 10.3390/molecules25051254

**Published:** 2020-03-10

**Authors:** Won-Gu Choi, Dong Kyun Kim, Yongho Shin, Ria Park, Yong-Yeon Cho, Joo Young Lee, Han Chang Kang, Hye Suk Lee

**Affiliations:** BK21 PLUS Team for Creative Leader Program for Pharmacomics-based Future Pharmacy, College of Pharmacy, The Catholic University of Korea, Bucheon 14662, Korea; cwg0222@catholic.ac.kr (W.-G.C.); kdk3124@catholic.ac.kr (D.K.K.); driger6103@catholic.ac.kr (Y.S.); hyacinthy7@catholic.ac.kr (R.P.); yongyeon@catholic.ac.kr (Y.-Y.C.); joolee@catholic.ac.kr (J.Y.L.); hckang@catholic.ac.kr (H.C.K.)

**Keywords:** doxorubicin, doxorubicinol, doxorubicinolone, doxorubicinone, 7-deoxydoxorubicinone, LC-MS/MS, mouse plasma

## Abstract

Doxorubicin, an anthracycline antitumor antibiotic, acts as a cancer treatment by interfering with the function of DNA. Herein, liquid chromatography-tandem mass spectrometry was for the first time developed and validated for the simultaneous determination of doxorubicin and its major metabolites doxorubicinol, doxorubicinone, doxorubicinolone, and 7-deoxydoxorubicinone in mouse plasma. The liquid–liquid extraction of a 10 μL mouse plasma sample with chloroform:methanol (4:1, *v*/*v*) and use of the selected reaction monitoring mode led to less matrix effect and better sensitivity. The lower limits of quantification levels were 0.5 ng/mL for doxorubicin, 0.1 ng/mL for doxorubicinol, and 0.01 ng/mL for doxorubicinone, doxorubicinolone, and 7-deoxydoxorubicinone. The standard curves were linear over the range of 0.5–200 ng/mL for doxorubicin; 0.1–200 ng/mL for doxorubicinol; and 0.01–50 ng/mL for doxorubicinone, doxorubicinolone, and 7-deoxydoxorubicinone in mouse plasma. The intra and inter-day relative standard deviation and relative errors for doxorubicin and its four metabolites at four quality control concentrations were 0.9–13.6% and –13.0% to 14.9%, respectively. This method was successfully applied to the pharmacokinetic study of doxorubicin and its metabolites after intravenous administration of doxorubicin at a dose of 1.3 mg/kg to female BALB/c nude mice.

## 1. Introduction

Doxorubicin (Adriamycin) is an anthracycline glycoside antitumor antibiotic used as a first-line drug in combination with other chemotherapy drugs for various types of cancers, including breast cancer, bladder cancer, soft tissue and bone sarcomas, malignant lymphoma, and acute lymphocytic leukemia [1]. However, it also has serious adverse effects: dose-dependent cardiotoxicity and myelosuppression [2,3]. Several nanotechnology-based doxorubicin preparations have been developed since the 1990s [3,4,5]. Some of these are commercially available, such as PEGylated liposomal Doxil^®^ and liposomal Myocet^®^, and others (e.g., micellar NK-911^®^, nanoparticles of Livatag^®^, and polymer–drug conjugates PK1 and PK2) are currently undergoing clinical trials [3,4].

**Figure 1:**
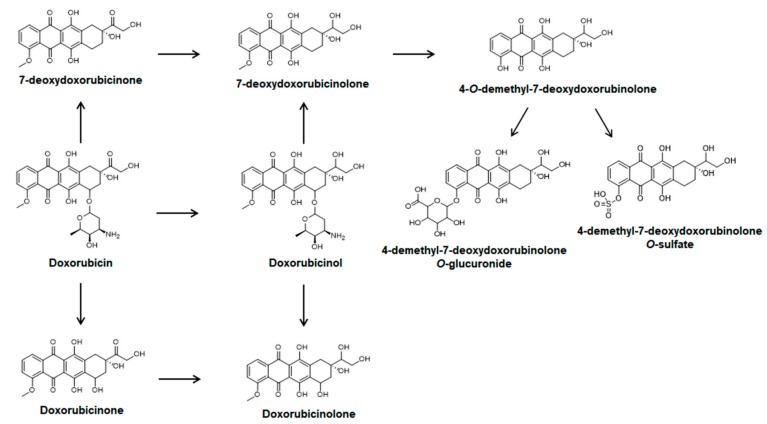
Metabolic pathways of doxorubicin.

Doxorubicin is metabolized to doxorubicinol, doxorubicinone, doxorubicinolone, 7-deoxydoxorubicinone, 7-deoxydoxorubicinolone, 4-*O-*demethyl-7-deoxydoxorubicinolone, 4-demethyl-7-deoxydoxorubicinolone sulfate, and 4-demethyl-7-deoxydoxorubicinolone glucuronide by carbonyl reduction, deglycosylation, *O*-demethylation, *O*-sulfation, and *O*-glucuronidation (Figure 1) [6,7,8,9,10]. Doxorubicinol, a major alcohol metabolite of doxorubicin formed by carbonyl reductases 1 and 3, is implicated in off-target cardiotoxicity of doxorubicin-treated patients [3,7,8,9,10].

High-performance liquid chromatography (HPLC) with fluorescence [11,12,13,14,15,16,17,18,19] or ultraviolet (UV) [20,21] detection, LC with mass spectrometry (LC-MS) [22,23] or tandem mass spectrometry (LC-MS/MS) [24,25,26,27,28,29,30,31,32,33,34,35,36], and capillary electrophoresis [37,38,39] methods have been used to analyze doxorubicin alone or with its metabolite doxorubicinol in various biological matrices, such as blood, serum, plasma, cells, and tissues. Protein precipitation with methanol, acetonitrile, or acetone [13,14,15,18,19,21,22,25,26,27,31,32,34,35]; liquid–liquid extraction with ethyl acetate or a mixture of chloroform and methanol [11,16,17,20,29,33]; and solid-phase extraction [23,24,28,30,36] methods have been used as sample clean-up procedures for a variety of biological samples. These methods resulted in lower limit of quantification (LLOQ) levels of 0.2–50 ng/mL for doxorubicin [11,12,13,14,15,16,17,18,19,20,21,22,23,24,25,26,27,28,29,30,31,32,33,34,35,36]; 0.5–1.25 ng/mL for doxorubicinol [11,23,25,27,28,30,33]; and 1–5 ng/mL for doxorubicinone, doxorubicinolone, 7-deoxydoxorubicinone, and 7-deoxydoxorubicinolone [11,12] using large volumes of blood, plasma, or serum (50–1000 μL). To date, no studies have used an LC–MS/MS method for the simultaneous determination of doxorubicin and its major four metabolites in plasma samples, but some have used HPLC with fluorescence detection [11,12].

We have developed, for the first time, a sensitive and rapid LC-MS/MS method for the simultaneous determination of doxorubicin and its major four metabolites, i.e., doxorubicinol, doxorubicinone, doxorubicinolone, and 7-deoxydoxorubicinone, using the least mouse plasma volume (10 μL) to evaluate the pharmacokinetics of doxorubicin and metabolites in formulation development and drug–drug interaction studies of doxorubicin. We successfully applied the method to characterize the pharmacokinetics of doxorubicin and its four metabolites after intravenous injection of doxorubicin at 1.3 mg/kg dose to female BALB/c nude mice.

## 2. Results

### 2.1. LC–MS/MS Analysis

To optimize the sensitive and simultaneous determination of doxorubicin and its four metabolites (i.e., doxorubicinol, doxorubicinone, doxorubicinolone, and 7-deoxydoxorubicinone) in a single run, various columns, such as Halo C18 (2.7 µm; 2.1 mm i.d. × 50 mm; Advanced Materials Technology, Wilmington, DE, USA), Acquity UPLC BEH C18 (1.7 µm; 2.1 mm i.d. × 50 mm; Milford, MA, USA), and Luna Omega C_18_ (1.6 µm; 2.1 mm i.d. × 50 mm; Phenomenex, Torrance, CA, USA) were assessed using gradient elution of methanol or acetonitrile and water with 0.1% formic acid as the mobile phase. The Luna Omega C18 column exhibited better separation, an excellent peak shape, and good sensitivity for the analytes using a gradient elution of 0.1% formic acid in 95% methanol and 0.1% formic acid in 0.1% formic acid in 5% methanol compared with the Halo C18 and Acquity BEH C18 columns. Using methanol instead of acetonitrile [19,20,21,22,23,24,25,26,27,28,29,30,31,32,33] as the strong eluent of the mobile phase resulted in increased ionization efficiency and linearity range for the five analytes. Daunorubicin, an analog of doxorubicin, was selected as and the internal standard (IS).

**Figure 2:**
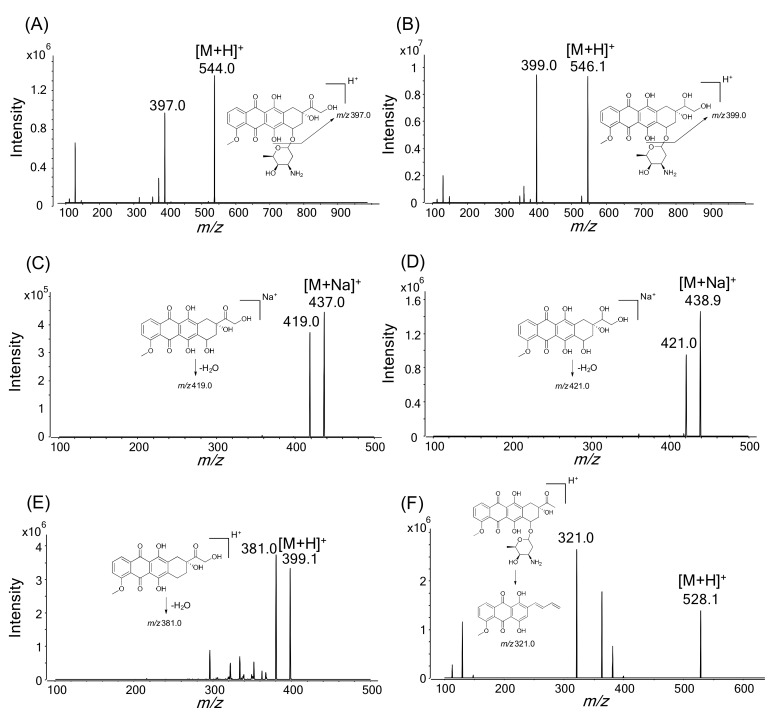
Product ion spectra of (**A**) doxorubicin, **(B**) doxorubicinol, (**C**) doxorubicinone, (**D**) doxorubicinolone, (**E**) 7-deoxydoxorubicinone, and (**F**) daunorubicin (internal standard).

The MS/MS parameters for all of the analytes were optimized using the flow-injection method to achieve maximum sensitivity, and selective reaction monitoring (SRM) transitions of the precursor ion ([M + H]^+^ or [M + Na]^+^) to the intense product ion were used for data acquisition due to the high selectivity and sensitivity (Figure 2). The positive ion mode yielded better sensitivity than the negative ion mode for all of the analytes.

**Figure 3:**
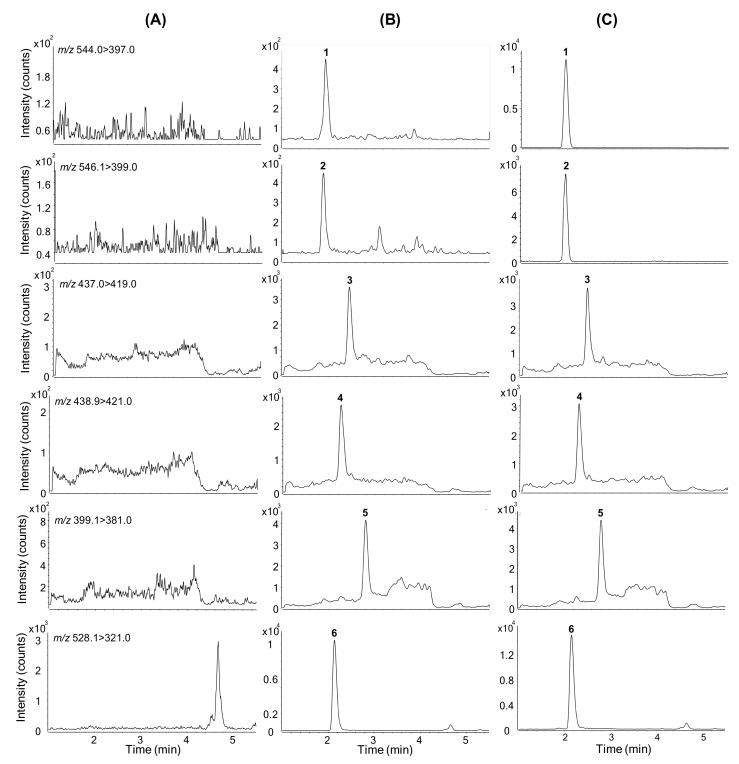
Selected reaction monitoring chromatograms of (**A**) mouse blank plasma: (**B**) Mouse plasma spiked with doxorubicin (0.5 ng/mL), doxorubicinol (0.1 ng/mL), doxorubicinone (0.01 ng/mL), doxorubicinolone (0.01 ng/mL), and 7-deoxydoxorubicinone (0.01 ng/mL) at the lower limit of quantification (LLOQ) levels and daunorubicin as the internal standard at 100 ng/mL; and (**C**) mouse plasma obtained 15 min after intravenous injection of doxorubicin at a dose of 1.3 mg/kg to a female BALB/c nude mouse. 1, Doxorubicin; 2, doxorubicinol; 3, doxorubicinone; 4, doxorubicinolone; 5, 7-deoxydoxorubicinone; 6, daunorubicin (internal standard).

No significant interference peaks were observed in the retention times of any of the analytes, indicating good method selectivity (Figure 3A). Figure 3B presents typical SRM chromatograms of the five analytes and IS in mouse plasma sample spiked with five analytes at LLOQ levels. The retention times were as follows: doxorubicin, 2.0 min; doxorubicinol, 1.9 min; doxorubicinone, 2.5 min; doxorubicinolone, 2.3 min; 7-deoxydoxorubicinone, 2.8 min; daunorubicin (IS), 2.2 min. Figure 3C presents representative SRM chromatograms of a plasma sample obtained 15 min after intravenous administration of doxorubicin in a mouse.

### 2.2. Method Validation

Calibration curves were linear over the concentration ranges of 0.5–200 ng/mL for doxorubicin; 0.1–200 ng/mL for doxorubicinol; and 0.01–50 ng/mL for doxorubicinone, doxorubicinolone, and 7-deoxydoxorubicinone in mouse plasma. The coefficients of determination were ≥ 0.9933 from linear regression analysis with a weighting of 1/concentration^2^ (Table 1). The relative standard deviation (RSD) and relative error (RE) of the calculated concentrations were less than 15% and ± 15%, respectively, for eight calibration points. The RSD values for the regression line slopes for the five analytes were ≤ 9.8%, indicating good method repeatability.

The intra and inter-day RSD and RE values for the five analytes in low, medium, and high quality control (QC) samples ranged from 0.9% to 10.9% and from −7.7% to 11.3%, respectively (Table 1), indicating that the accuracy and precision of this method are acceptable. The intra and inter-day RSD and RE values for diluted QC plasma samples of the five analytes with dilution factor of 50 ranged from 5.6% to 13.0% and from –9.0% to 8.0%, respectively (Table 1), supporting good dilution integrity.

The LLOQ values for doxorubicin, doxorubicinol, doxorubicinone, doxorubicinolone, and 7-deoxydoxorubicinone were 0.5, 0.1, 0.01, 0.01, and 0.01 ng/mL, respectively, in mouse plasma (Table 1). The RE and RSD values of the five analytes at the LLOQ QC levels were within the criteria of RE (±20%) and RSD (20%); i.e., –13.0% to 14.9% and 4.5% to 13.6%, respectively (Table 1). The limit of detection (LOD) values for doxorubicin, doxorubicinol, doxorubicinone, doxorubicinolone, and 7-deoxydoxorubicinone were 0.26, 0.06, 0.006, 0.007, and 0.006 ng/mL, respectively (Table 1).

Using liquid−liquid extraction with chloroform:methanol (4:1, *v*/*v*), the recoveries of doxorubicin, doxorubicinol, doxorubicinone, doxorubicinolone, 7-deoxydoxorubicinone, and daunorubicin (IS) in mouse plasma were 81.7–86.4, 84.1–87.9, 77.0–90.4, 81.6–94.7, 81.3–87.7, and 108.8%, respectively (Table 2). The matrix effects for doxorubicin, doxorubicinol, doxorubicinone, doxorubicinolone, 7-deoxydoxorubicinone, and daunorubicin (IS) were 112.9–119.7%, 94.8–109.6%, 105.1–117.9%, 99.0–111.0%, 98.3–108.3%, and 88.0%, respectively, with RSD ≤ 14.2% at low, medium, and high QC levels (Table 2), indicating the presence of a small matrix effect. Liquid−liquid extraction using chloroform:methanol (4:1, *v*/*v*) resulted in higher sensitivity and smaller matrix effects for doxorubicin and its four metabolites compared with the protein precipitation method using methanol, acetonitrile, or acetone [13,14,15,18,19,21,22,25,26,27,31,32,34,35]. Our method yielded LLOQ levels of doxorubicin (0.5 ng/mL) and its major four metabolites (0.1–0.01 ng/mL) that were better than those reported [11,12,13,14,15,16,17,18,19,20,21,22,23,24,25,26,27,28,29,30,31,32,33,34,35,36] using the least plasma volume of 10 μL.

Table 3 presents three freeze–thaw, short-term storage on ice, and post-preparation stabilities of each analyte; these processes had negligible effects on sample stability.

Incurred sample reanalysis (ISR) was performed by reanalyzing 10 mouse plasma samples obtained from the pharmacokinetic study in mice. The percentage differences between ISR and the original concentration values for all of the repeat samples were within ±20%, indicating ISR is successful and this method is reproducible.

### 2.3. Pharmacokinetics of Doxorubicin in Mice

**Figure 4:**
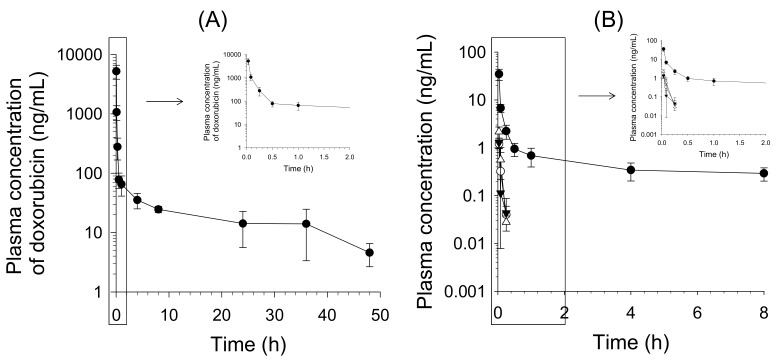
Mean plasma concentration–time profiles of (**A**) doxorubicin and (**B**) the four metabolites doxorubicinol (⬤), doxorubicinone (◯), doxorubicinolone (▼), and 7-deoxydoxorubicinone (△) after a single intravenous injection of doxorubicin at a dose of 1.3 mg/kg to female BALB/c nude mice. Points represent means ± SDs (*n* = 5). The inset figures represent the plasma concentration–time curves of five analytes from 0 h to 2 h.

Figure 4 presents the mean plasma concentration–time profiles of doxorubicin and its four metabolites following an intravenous injection of doxorubicin at a dose of 1.3 mg/kg to female BALB/c nude mice. The mean plasma concentration–time profiles of doxorubicinol, doxorubicinone, doxorubicinolone, and 7-deoxydoxorubicinone, the major metabolites of doxorubicin (Figure 1) [6,7,8,9,10], were determined and compared to that of unchanged doxorubicin in plasma, after intravenous injection of doxorubicin to mice (Figure 4, Table 4). The maximum plasma concentrations (C_max_) of doxorubicinol, doxorubicinone, doxorubicinolone, and 7-deoxydoxorubicinone were 34.7 ± 9.3, 1.2 ± 0.3, 1.3 ± 0.3, and 2.2 ± 0.6 ng/mL, respectively, at the first sampling point (2 min), supporting the rapid metabolism of doxorubicin. However, the plasma concentrations of doxorubicinone, doxorubicinolone, and 7-deoxydoxorubicinone were below the LLOQ (0.01 ng/mL) from 0.5 h after injection of doxorubicin (Figure 4), and therefore, other pharmacokinetic parameters of doxorubicinone, doxorubicinolone, and 7-deoxydoxorubicinone could not be calculated. Table 4 lists the pharmacokinetic parameters of doxorubicin and a major and active metabolite, doxorubicinol. The half-life of the primary metabolite doxorubicinol (5.4 h) was shorter than that of doxorubicin (15.3 h), and the area under the concentration–time curve to last time (AUC_last_) of doxorubicinol was 0.38% of that of doxorubicin.

## 3. Materials and Methods

### 3.1. Materials

Doxorubicin (purity, 95.0%) was obtained from MedKoo Biosciences (Morrisville, NC, USA). Doxorubicinol (purity, 90.0%), doxorubicinone (purity, 98.0%), doxorubicinolone (purity, 90.0%), 7-deoxydoxorubicinone (purity, 95.0%), and daunorubicin hydrochloride (purity, 96%) were obtained from Toronto Research Chemicals Inc. (Toronto, ON, Canada). Dimethyl sulfoxide, chloroform, and formic acid were purchased from Sigma–Aldrich Co. (St. Louis, MO, USA). Water, acetonitrile, and methanol (LC-MS grade) were supplied by Fisher Scientific Co. (Fair Lawn, NJ, USA). All other chemicals used were of the highest quality available.

### 3.2. Preparation of Calibration Standards and Quality Control Samples

Each standard stock solution was prepared separately by dissolving doxorubicin, doxorubicinol, doxorubicinone, doxorubicinolone, and 7-deoxydoxorubicinone (1 mg each) in 1 mL of dimethyl sulfoxide. Mixed working standard solutions of the five analytes were prepared by mixing each working standard stock solution with methanol. IS working solution (daunorubicin hydrochloride, 100 ng/mL) was prepared by diluting an aliquot of the stock solution with methanol. All standard solutions were stored at 4 °C in darkness for 4 weeks.

Mouse plasma calibration standards were prepared at eight concentration levels: 0.5, 1, 2, 5, 25, 100, 160, and 200 ng/mL for doxorubicin; 0.1, 0.2, 1, 5, 25, 100, 160, and 200 ng/mL for doxorubicinol; and 0.01, 0.02, 0.1, 0.5, 2.5, 10, 40, and 50 ng/mL for doxorubicinone, doxorubicinolone, and 7-deoxydoxorubicinone. QC samples were prepared at the concentrations of 1.5, 20, and 150 ng/mL for doxorubicin; 0.3, 10, and 150 ng/mL for doxorubicinol; and 0.03, 1, and 37.5 ng/mL for doxorubicinone, doxorubicinolone, and 7-deoxydoxorubicinone in drug-free mouse plasma and stored at −80 °C until analyzed.

### 3.3. Sample Preparation

The 10 µL aliquots of blank mouse plasma, calibration standards, and QC samples were stored on ice, and then vortex-mixed with 4 µL of daunorubicin (IS) in methanol (100 ng/mL), 30 µL of 50 mM potassium phosphate buffer (pH 7.4), and 400 µL of chloroform:methanol (4:1, *v*/*v*) for 10 min. Following centrifugation at 10,000 *g* for 10 min, 300 µL of the supernatant was transferred into a new amber polypropylene tube. The organic layer was evaporated to dryness at 35 °C over 10 min using a vacuum evaporator (EZ-2 plus, Genevac Ltd., Ipswich, UK). The residues were dissolved in 30 µL of 0.1% formic acid in 40% methanol and centrifuged. An aliquot (3 µL) was injected onto the LC-MS/MS system for analysis.

### 3.4. LC-MS/MS Analysis

An ultra-performance liquid chromatograph (Agilent 1290; Agilent Technologies, Wilmington, DE, USA) coupled with a tandem mass spectrometer (Agilent 6495) was used for the LC-MS/MS analysis. Chromatographic separation was performed on a Luna Omega C18 column (1.6 µm; 2.1 mm i.d. × 50 mm, Phenomenex, Torrance, CA, USA) using a gradient elution of 0.1% formic acid in 5% methanol (MP A) and 0.1% formic acid in 95% methanol (MP B) at a flow rate of 0.2 mL/min as follows: 40% MP B for 0.25 min, 40% to 70% MP B for 0.25 min, 70% to 85% MP B for 2 min, 85% to 98% MP B for 0.1 min, 98% MP B for 2.9 min, 98% to 40% MP B for 0.1 min, and 40% MP B for 2.4 min. The column and autosampler tray were maintained at 35 °C and 4 °C, respectively. The electrospray source settings for ionization of the analytes in positive mode were as follows: gas temperature, 260 °C; gas flow, 11 L/min; nebulizer, 30 psi; sheath gas temperature, 400 °C; sheath gas flow, 12 L/min; capillary voltage, 4000 V; and nozzle voltage, 2000 V. Nitrogen gas was used as the collision gas at a pressure of 2 bar on the instrument. The collision energies for the fragmentation of doxorubicin, doxorubicinol, doxorubicinone, doxorubicinolone, 7-deoxydoxorubicinone, and daunorubicin (IS) were 9, 10, 12, 14, 22, and 30 eV, respectively. The SRM transitions for the quantification were as follows: *m/z* 544.0→397.0 for doxorubicin; *m/z* 546.1→399.0 for doxorubicinol; *m*/*z* 437.0→419.0 for doxorubicinone; *m*/*z* 438.9→421.0 for doxorubicinolone; *m*/*z* 399.1→381.0 for 7-deoxydoxorubicinone; and *m*/*z* 528.1→321.0 for daunorubicin. Mass Hunter software (Version B.07.00, Agilent Technologies, Wilmington, DE, USA) was used for LC-MS/MS system control and data processing.

### 3.5. Method Validation

Method validation was performed according to the methods set out in the FDA Guidance on Bioanalytical Method Validation. To evaluate intra and inter-day precisions and accuracies, we analyzed batches of calibration standards and QC samples in five replicates on three different days as follows: 1.5, 20, and 150 ng/mL for doxorubicin; 0.3, 10, and 150 ng/mL for doxorubicinol; and 0.03, 1, and 37.5 ng/mL for doxorubicinone, doxorubicinolone, and 7-deoxydoxorubicinone. Accuracy was defined as the RE (%) of the measured mean value deviating from the nominal value, and precision was defined as the RSD (%) of the measured concentration.

To evaluate the dilution integrity, mouse plasma samples spiked with 1500 ng/mL of doxorubicin and doxorubicinol; and 375 ng/mL of doxorubicinone, doxorubicinolone, and 7-deoxydoxorubicinone—which were diluted with pooled blank mouse plasma at dilution factors of 50 in five replicates and analyzed for three successive days.

The LLOQ value was defined as the lowest amount of each analyte in a mouse plasma sample that could be quantified as follows: signal-to-noise ratio > 5; RSD < ± 20%; RE < 20%. Each LOD was calculated as 3δ/s, where δ is the standard deviation of the response near an expected LOD concentration (*n* = 7) and s is the slope of the calibration curve [40].

The stability of each of the five analytes in mouse plasma was evaluated by analyzing low and high QC samples in triplicate: post-preparation sample stability in the autosampler at 4 °C for 24 h; short-term storage stability following storage of plasma samples on ice for 2 h; and three freeze–thaw cycles.

The matrix effect for each analyte was assessed by comparing the peak areas of the analytes spiked after extraction into blank plasma extracts originating from six different mice to the mean peak areas for neat solutions of the analytes at three concentration levels. The recoveries of each analyte were determined by comparing the peak areas of the extract of analyte-spiked plasma with those of the analytes spiked post-extraction into six different blank plasma extracts at three concentration levels.

ISR was performed by reanalysis of 10 mouse plasma samples obtained from the pharmacokinetic study of doxorubicin in mice, where sample numbers were equivalent to 10% of the study sample size. The percentage difference of the results between the original analysis and the repeat analysis was determined with the following equation:

(1) $$\frac{\left(\mathrm{repeat}\;-\;\mathrm{original}\right)\;\times \;100}{\mathrm{mean}}$$

### 3.6. Pharmacokinetic Study of Doxorubicin in Mice

This validated method was applied to the pharmacokinetic study of doxorubicin and its metabolites after a single intravenous injection of doxorubicin at a dose of 1.3 mg/kg to BALB/c nude female mice (*n* = 5; body weights, 16.4–18.2 g; Orient Bio, Seongnam, Korea). The study protocol was approved by the Institutional Animal Care and Use Committee at the Catholic University of Korea (approval number 2019-036-01). Animals were kept in plastic cages with unlimited access to standard a mouse diet (Orient Bio) and water before the experiment, and were maintained at a temperature of 23 ± 2 °C, a 12 h light/dark cycle, and a relative humidity of 50% ± 10%. Doxorubicin hydrochloride was dissolved in normal saline and administered to the tail vein of the mice at a dose of 1.3 mg/kg. A blood sample (approximately 40 µL) was collected from the retro-orbital plexus of an individual mouse under light anesthesia with isoflurane at 2, 5, 15, and 30 min, and at 1, 4, 8, 24, 36, and 48 h after drug administration. Plasma samples were harvested by centrifugation at 10,000 *g* for 5 min at 4 °C; 10 µL plasma samples were immediately collected in 1.5-mL amber polypropylene microcentrifuge tubes and stored at –80 °C until LC-MS/MS analysis.

Pharmacokinetic parameters, including the area under the plasma concentration–time curve during the period of observation (AUC_last_), AUC to infinite time (AUC_inf_), the terminal half-life (t_1/2_), clearance (CL), volume of distribution at steady state (V_ss_), and mean residence time (MRT), were evaluated using noncompartmental analysis (WinNonlin; Pharsight, Mountain View, CA, USA). The maximum plasma concentration (C_max_) and the time to reach C_max_ (T_max_) of the metabolites were directly obtained from the experimental data. Each value is expressed as the mean ± standard deviation (SD).

## 4. Conclusions

A sensitive and rapid LC-MS/MS method using liquid–liquid extraction as a sample clean-up procedure was for the first time developed and validated for the simultaneous determination of doxorubicin and its major four metabolites—doxorubicinol, doxorubicinone, doxorubicinolone, and 7-deoxydoxorubicinone—with LLOQ levels of 0.5, 0.1, 0.01, 0.01, and 0.01 ng/mL, respectively, in 10 μL of mouse plasma. This method was successfully applied to the pharmacokinetics study of doxorubicin and its four metabolites after intravenous administration of doxorubicin at 1.3 mg/kg dose to BALB/c female nude mice.

## Figures and Tables

**Table 1:** Linearity, limit of detection (LOD), LLOQ, intra and inter-day accuracies (relative error (RE), %), and precision (relative standard deviation (RSD), %) of doxorubicin, doxorubicinol, doxorubicinone, doxorubicinolone, and 7-deoxydoxorubicinone in mouse plasma QC samples.

Analyte	Concentration Range (ng/mL), Linear Equation,^a^ Linearity (r^2^),^b^ LLOQ (ng/mL), LOD (ng/mL)	QC concentration (ng/mL)	Intraday (*n* = 5)		Interday (*n* = 15)	
			RE (%)	RSD (%)	RE (%)	(RSD %)
Doxorubicin	0.5–200	0.5	−6.6	6.3	−1.7	8.2
	*y* = 0.002380*x* + 0.002187	1.5	−6.9	7.5	1.7	9.4
	0.9933	20.0	−7.7	5.7	−0.8	8.9
	0.5	150	4.5	4.3	5.8	8.1
	0.26	1500	−6.8	13.1	−4.7	10.3
Doxorubicinol	0.1–200	0.1	14.9	4.5	4.0	12.6
	*y* = 0.03629*x* − 0.003557	0.3	11.3	6.4	5.8	9.5
	0.9973	10.0	4.7	0.9	8.5	5.6
	0.1	150	6.9	2.0	6.3	6.0
	0.06	1500	8.0	5.6	5.4	6.1
Doxorubicinone	0.01–50	0.01	3.3	11.9	−2.3	13.6
	*y* = 0.002878*x* + 0.009975	0.03	7.4	5.7	−1.3	9.2
	0.9965	1.0	1.3	9.8	−2.1	9.6
	0.01	37.5	5.8	3.6	1.1	8.2
	0.006	375	−3.0	7.4	−0.9	6.9
Doxorubicinolone	0.01–50	0.01	1.3	6.3	−3.7	7.7
	*y* = 0.002539*x* + 0.009896	0.03	8.9	3.7	0.7	10.9
	0.9965	1.0	7.0	7.3	7.0	8.2
	0.01	37.5	9.8	1.6	10.2	4.0
	0.007	375	−5.1	5.7	−3.2	6.3
7-Deoxydoxorubicinone	0.01–50	0.01	−13.0	12.2	−6.6	11.1
	*y* = 0.02980*x* + 0.009917	0.03	6.0	7.4	0.9	7.5
	0.9962	1.0	−3.6	2.5	0.1	6.3
	0.01	37.5	1.7	9.8	3.0	6.6
	0.006	375	−9.0	6.7	1.3	7.9

^a^*y*: Peak area ratio, *x*: concentration; ^b^*r*^2^: coefficients of determination.

**Table 2:** Matrix effects and recoveries of doxorubicin, doxorubicinol, doxorubicinone, doxorubicinolone, 7-deoxydoxorubicinone, and daunorubicin (internal standard) using six different mouse plasma samples (*n* = 6).

Compound	Nominal Concentration (ng/mL)	Matrix Effect^a^ (%)		Recovery^b^ (mean ± SD, %)
		Mean	RSD (%)	
Doxorubicin	1.5	112.9	3.6	85.3 ± 8.6
	20	119.7	11.1	86.4 ± 10.6
	150	114.5	6.2	81.7 ± 7.0
Doxorubicinol	0.3	94.8	1.6	84.1 ± 4.8
	7.5	109.6	9.4	85.4 ± 9.5
	150	108.3	7.2	87.9 ± 7.5
Doxorubicinone	0.03	117.9	14.2	77.0 ± 4.9
	1	108.1	7.8	90.4 ± 6.8
	37.5	105.1	5.2	86.5 ± 8.6
Doxorubicinolone	0.03	111.0	12.1	81.6 ± 6.1
	1	99.0	7.0	94.7 ± 3.9
	37.5	105.8	3.6	89.1 ± 6.4
7-Deoxydoxorubicinone	0.03	98.3	12.0	81.3 ± 5.4
	1	98.8	10.7	82.8 ± 4.8
	37.5	108.3	6.5	87.7 ± 9.4
Daunorubicin	100	88.0	9.5	108.8 ± 6.7

^a^ Matrix effect was calculated as (peak area of each analyte spiked post-extraction of each blank plasma/mean peak area of the equivalent analyte standard solution) × 100. ^b^ Recovery was calculated as (peak area of an analyte-spiked plasma prior to liquid−liquid extraction/peak area of an analyte spiked after liquid–liquid extraction of blank plasma) × 100.

**Table 3:** Post-preparation, short-term, and freeze–thaw stabilities of doxorubicin, doxorubicinol, doxorubicinone, doxorubicinolone, and 7-deoxydoxorubicinone in mouse plasma QC samples (n = 3).

Analytes and Nominal Concentrations (ng/mL)	Post-Preparation (24 h at 4 °C)		Short-Term (2 h on ice)		Freeze–Thaw (Three Cycles of −80 °C to Room Temperature)	
	RE, %	RSD, %	RE, %	RSD, %	RE, %	RSD, %
Doxorubicin						
1.5	4.7	5.1	0.0	10.9	−2.7	11.5
150	7.6	6.4	−4.9	10.4	4.3	2.6
Doxorubicinol						
0.3	−6.9	12.0	13.6	6.9	0.1	10.6
150	6.9	3.8	−9.3	1.1	3.8	7.4
Doxorubicinone						
0.03	−8.0	7.5	5.0	2.9	−9.7	4.6
37.5	3.3	5.6	0.2	10.2	5.9	2.7
Doxorubicinolone						
0.03	−4.3	9.6	10.5	4.2	2.9	6.2
37.5	12.2	6.5	3.4	5.9	5.7	1.9
7-Deoxydoxorubicinone						
0.03	1.3	5.7	−5.2	13.9	−5.0	3.7
37.5	12.2	5.4	−0.8	9.8	1.7	2.8

**Table 4:** Pharmacokinetic parameters of doxorubicin and its metabolite doxorubicinol after a single intravenous injection of doxorubicin at a dose of 1.3 mg/kg to female BALB/c nude mice. Data are shown as means ± SDs (*n* = 5).

Pharmacokinetic Parameters	Doxorubicin	Doxorubicinol
Area under the concentration–time curve to last time (AUC_last_, ng∙h/mL)	1415.6 ± 303.6	5.4 ± 1.1
AUC to infinite time (AUC_inf_, ng∙h/mL)	1475.5 ± 392.8	7.6 ± 2.4
Clearance (CL, mL/h/kg)	896 ± 167	–
Volume of distribution at steady state (Vss, mL/kg)	12,636 ± 3410	–
Half-life (t_1/2_, h)	15.3 ± 3.4	5.4 ± 3.2
Mean residence time (MRT, h)	9.4 ± 1.8	–
Maximum plasma concentration (C_max_, ng/mL)	–	34.7 ± 9.3
Time to reach C_max_ (T_max_, h)	–	0.033
